# Weed Dynamics during Transition to Conservation Agriculture in Western Kenya Maize Production

**DOI:** 10.1371/journal.pone.0133976

**Published:** 2015-08-03

**Authors:** Judith A. Odhiambo, Urszula Norton, Dennis Ashilenje, Emmanuel C. Omondi, Jay B. Norton

**Affiliations:** 1 Department of Plant Sciences, University of Wyoming, Dept. 3354, 1000 E. University Ave., Laramie, WY 82071, United States of America; 2 Program in Ecology, University of Wyoming, Laramie, WY 82071, United States of America; 3 Manor House Agricultural Centre, Private Bag, Kitale, 30200, Kenya; 4 Department of Ecosystem Science and Management, Dept. 3354, 1000 E. University Ave., Laramie, WY 82071, United States of America; Instituto de Agricultura Sostenible (CSIC), SPAIN

## Abstract

Weed competition is a significant problem in maize (*Zea mays*, L.) production in Sub-Saharan Africa. Better understanding of weed management and costs in maize intercropped with beans (*Phaseolus vulgaris*, L.) during transition to conservation agricultural systems is needed. Changes in weed population and maize growth were assessed for a period of three years at Bungoma where crops are grown twice per year and at Trans-Nzoia where crops are grown once per year. Treatments included three tillage practices: minimum (MT), no-till (NT) and conventional (CT) applied to three cropping systems: continuous maize/bean intercropping (TYPICAL), maize/bean intercropping with relayed mucuna after bean harvest (RELAY) and maize, bean and mucuna planted in a strip intercropping arrangement (STRIP). Herbicides were used in NT, shallow hand hoeing and herbicides were used in MT and deep hoeing with no herbicides were used in CT. Weed and maize performance in the maize phase of each cropping system were assessed at both locations and costs of weed control were estimated at Manor House only. Weed density of grass and forb species declined significantly under MT and NT at Manor House and of grass species only at Mabanga. The greatest declines of more than 50% were observed as early as within one year of the transition to MT and NT in STRIP and TYPICAL cropping systems at Manor House. Transitioning to conservation based systems resulted in a decline of four out of five most dominant weed species. At the same time, no negative impact of MT or NT on maize growth was observed. Corresponding costs of weed management were reduced by $148.40 ha^-1^ in MT and $149.60 ha^-1^ in NT compared with CT. In conclusion, farmers can benefit from effective and less expensive weed management alternatives early in the process of transitioning to reduced tillage operations.

## Introduction

Smallholder farmers in Sub-Saharan Africa (SSA) grow maize (*Zea mays*, L.) intercropped with beans (*Phaseolus vulgaris*, L.) every year. They use animal-drawn plows and hand hoes to invert the soil. Frequent deep tillage however, causes significant declines in soil fertility and consequently, crop yields [1, 2]. Weed competition with crops is also a very serious problem in SSA and using tillage for weed control has not been very effective [3, 4].

A number of conservation agriculture (CA) practices designed to replace or improve continuous maize/bean intercropping in the region intend to introduce nitrogen (N) fixing cover crops, reduce soil disturbance and retain surface crop residues [5]. Ultimately, these practices should improve crop yields [6], reduce costs of crop production [7], increase soil organic matter (SOM) content and improve long-term soil health [8]. However, adoption of CA is often hindered by farmers’ limited understanding of the changes in weed control practices and crop performance during the transition period [9].

**Fig 1:**
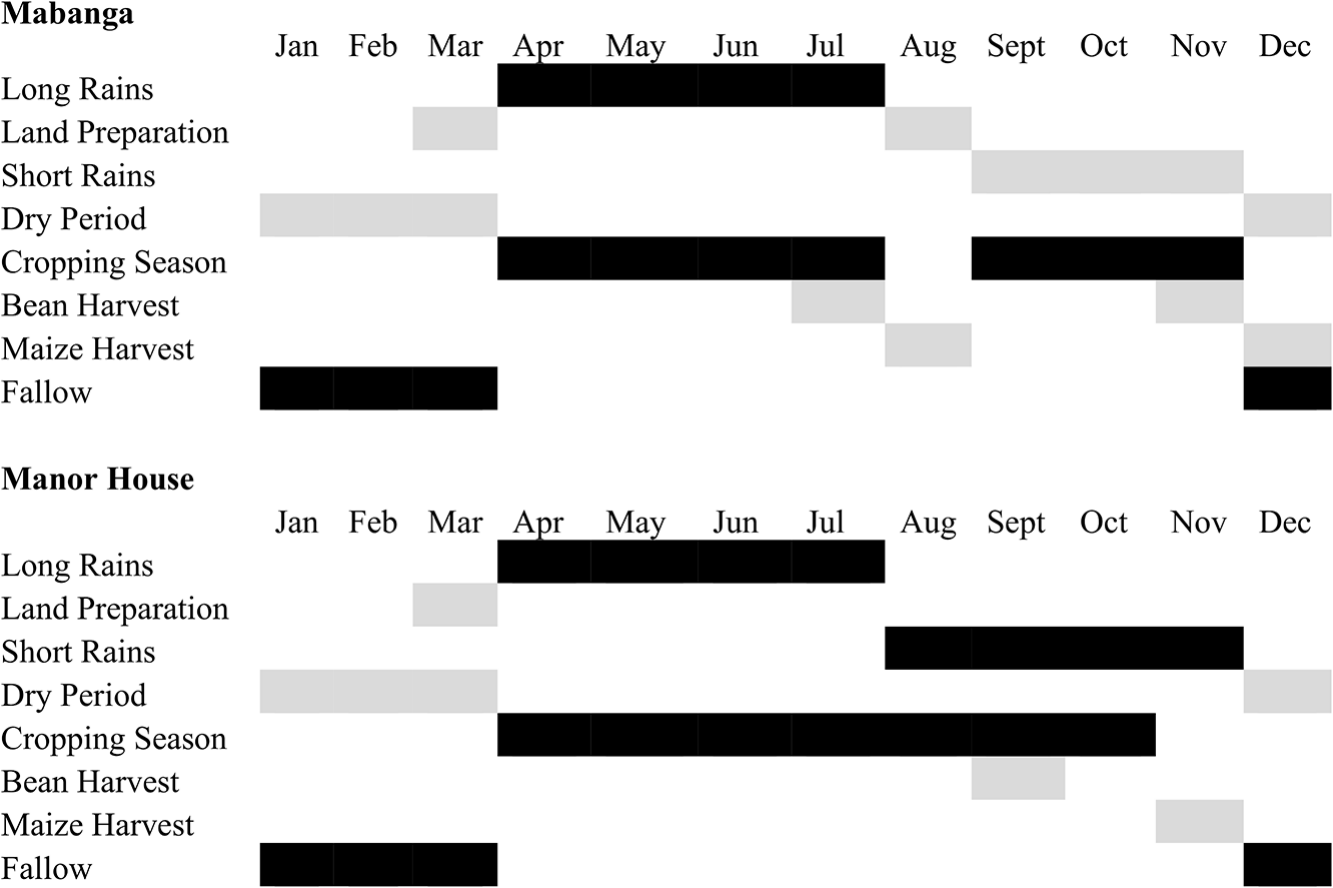
Annual crop and field management practices associated with two growing seasons (Mabanga) and one growing season (Manor House).

Western Kenya has a bimodal pattern of precipitation characterized by the long and short rainy seasons (Fig 1). Crops are grown during one long growing season in the cooler, higher-elevation region and during two growing seasons in the warmer, lower-elevation region. Growing crops during two growing seasons however, necessitates more frequent tillage and results in shorter periods of soil rest [10].

Farmers recognize the benefits of reducing tillage but remain uncertain about managing weeds while converting from mechanical to chemical weed control. Better understanding of weed population changes during transition from frequently tilled continuous maize/bean intercropping to reduced or no-till CA practices can therefore, ensure successful adoption [11]. It is known that transition to reduced tillage practices without using herbicides has resulted in high weed densities and reduced crop yields in some areas of SSA [12]. Farmers often perceive the use of chemical weed control as cost prohibitive and they lack training on herbicide application [13]. In addition, effects of leguminous cover crops on weed species assemblages are unclear. For example, velvet bean, also known as mucuna (*Mucuna pruriens* (L.) DC.) is successfully used in other parts of the world. Not only does it fix atmospheric N, but its rapidly growing biomass is capable of suppressing weeds [14–16] and stimulating suicidal germination of the purple witchweed, also known as striga (*Striga hermonthica*), a parasite that targets maize [17].

This project is a part of a larger study assessing alternative CA practices that incorporate reducing tillage and introducing cover crops. The main objective was to evaluate the effects of transition from conventional maize/bean intercropping to CA tillage and cropping systems on weed population, maize growth and management costs. We hypothesized that reducing tillage and rotating maize/bean intercropping with mucuna cover crop result in declines in weed density and diversity with no negative impact on maize performance and reduction in weed management costs.

## Materials and Methods

### Site Characteristics

The study was conducted for three years (2011 to 2013) at two sites in western Kenya. Manor House Agricultural Center (Manor House) in Trans-Nzoia County is located at 1,890 meters elevation, at 010°01^′^ N LAT and, 35° 00^′^ E LONG in the upper midland agro-ecological zone [18]. The site receives 1,300 mm of rainfall annually with mean average air temperature of 20°C [19]. Mabanga Farmers Training Center (Mabanga) in Bungoma County is located at 1,433 meters elevation, at 00°35^′^N LAT and 34°34^′^E LONG in the lower midland agro-ecological zone [18]. The site receives 1,100 mm of rainfall annually with mean average temperature of 23°C [19]. The two locations experience bimodal rainfall with a long rainy season between March and August and a short rainy season between September and November (Fig 1). Farmers in Trans-Nzoia grow crops during one long cropping season that spans both the long and short rainy seasons. In Bungoma average temperatures are 3°C higher than in Trans-Nzoia, which allows farmers to grow crops twice per year during long and short rainy seasons. Soils at both locations are Ferrasols with high contents of kaolinite clay and low pH [20]. Soils at Manor House are classified as sandy clay loams and soils at Mabanga are clay loams. Conventionally tilled continuous maize/bean production was practiced at both locations before treatment establishment.

### Study Design

A two-way factorial experiment was designed to compare three tillage management systems across three cropping rotations. The experimental layout was a split-plot randomized complete block design (RCBD) replicated four times. Each plot measured 10 m by 10 m with a 1-m border in between. The main factor was tillage applied at three levels: conventional tillage (CT), minimum tillage (MT) and no-till (NT). The second factor was cropping rotation applied at three levels: typical farmers cropping practice of repetitive maize/bean intercropping (TYPICAL), maize/bean intercropping with mucuna as a relay intercropped with maize after bean harvest (RELAY) and strip intercropping rotation system that consisted of maize, bean and mucuna planted in separate narrow strips on the same plot and rotating annually in a maize/bean/mucuna sequence (STRIP). Each strip in STRIP cropping measured 10 m by 3 m (four maize rows). Tillage in CT consisted of land preparation using inversion-type moldboard plowing (two times), followed by harrowing and planting using a hand hoe. Weeding was performed two times per season using a hand hoe. Depth of hoeing was typically 18–20 cm during field preparation and down to 10–15 cm during weeding. Tillage in MT was done using shallow hand hoeing to about 5-cm depth and performed once during land preparation and once during weeding. No tillage operations were performed in NT and opening soil for seed planting was done using a large machete called a “panga”.

Selection of crop varieties for the two locations was based on recommendations from Kenya Seed Company Ltd. Maize H6213 hybrid was planted at Manor House and H513 hybrid was planted at Mabanga. Common bean Rosecoco-GLP2 and white seed mucuna were planted at both sites. Planting for one growing season in Manor House and for the long rainy season at Mabanga was done in mid-March (Fig 1). Mucuna was planted in mid-March in STRIP and after bean harvest in RELAY, which occurred in July at Mabanga and August at Manor House. Maize was planted every 0.3 m with row spacing of 0.75 m. Beans in TYPICAL and RELAY cropping were planted at a spacing of 0.15 m between bean plants in maize inter-rows. Beans in STRIP cropping were planted at a spacing of 0.15 m between bean plants with row spacing of 0.38 m. Mucuna was planted at a 0.5-m within-row spacing, with planting in maize inter-rows in RELAY and with 0.75-m row spacing in STRIP. Mucuna residue remained on soil surface in NT and was incorporated to soil in MT and CT during tillage operations. Maize was fertilized with 57 kg of phosphate ha^-1^ as di-ammonium phosphate (DAP) that also delivered 19.0 kg N ha^-1^ at planting. Additional N was top-dressed at 37.5 kg N ha^-1^ as calcium ammonium nitrate (CAN). Beans received 60 kg phosphate ha^-1^ as single super-phosphate (SSP) at planting time.

Herbicides recommended for maize and beans were used in MT and NT only. One week before planting in March, the non-selective pre-emergence herbicide, S-metolachlor [(Dual Gold), 2-chloro-*N*- (2-ethyl-6-methylphenyl)-*N*- (2-methoxy-1-methylethyl) acetamide] and glyphosate [(Touchdown), (*N*-phosphonomethyl) glycine)] were applied using a hand operated backpack sprayer at the rate of 576 g and 750 g ha^-1^, respectively. The post-emergence herbicide, bentazone [(Basagran), (3-methylehyl)-1H-2, 3-Benzothiadiazin-4(3H)-one 2, 2-dioxide] was also applied using a hand operated backpack sprayer at the rate of 600 g ha^-1^ in NT when maize and bean plants had two to three fully developed leaves.

### Weed Measurements

All measurements of plant parameters were taken annually at each location eight weeks after planting (two weeks after the last weeding in CP and MT). At that time, maize plants had seven to eight fully developed leaves (V7-V8 growth stage). This stage of maize development can be most vulnerable to competition with weeds and hence, establishment of maize yield potential [21]. All individual plants were counted to assess plant cover using a 1.0- by 0.5-m frame placed at four random locations. Plants were divided to two groups: grasses and forbs. At Manor House, all plants were identified and Shannon Diversity Index was calculated for weeds present in field in 2012 and 2013. The Shannon Diversity Index (H') was calculated based on the formula developed by Magurran [22].

$$H'=-\sum_{i=1}^{s}\frac{ni}{N}\;\text{x log}\frac{ni}{N}$$ (1)

Where:

s = number of species present

n*i* = total number of individuals of the i^th^ species

N = total number of all individual species

### Cost of Weed Control

Weed management input costs for different tillage practices were assessed at Manor House only and comprised of actual costs obtained from Kenya Seed Company Ltd. Costs associated with manual labor were based on man-hours recorded for specific operations and costs per hour were derived from typical labor costs for the region. Costs of contracting animal-drawn operations and equipment rental also included fuel and labor and were based on actual costs incurred.

### Maize Growth

Maize height and leaf area (LA) were determined at Manor House only on five randomly selected plants also at V7-V8 maize vegetative growth stage. Plant height was assessed using a measuring tape stretched between the plant base at the soil surface and the arch of the uppermost fully developed leaf. The number of fully developed leaves on each plant was counted and leaf length and leaf width were measured and recorded. Leaf length was measured from the junction of the leaf blade collar to the leaf sheath tip and leaf width was measured from edge to edge at the widest part of the leaf. Leaf area (LA) was calculated using the equation from Mokhtarpour et al. [23].

LA=C(LxW) (2)

Where:

LA = leaf area (cm^-2^)

L = leaf length (cm)

W = leaf maximum width (cm)

C = 0.75 (correction factor calculated by Aikins et al.[24])

Leaf area index (LAI) was calculated as the sum of LAs of all fully developed leaves on individual plants per meter square of ground area (m^2^ m^-2^) [25]. Leaf N concentration (LNC) was determined on the lowest fully developed leaf at V7-8 stage. The leaf was cut at the base, oven dried at 65°C and finely ground. Approximately five grams were wrapped in a 5- x 9-mm tin capsule and analyzed by dry combustion using Carlo Erba combustion on EA 1100 C/N Analyzer (Carlo Erba Instruments, Milan Italy).

### Statistical Analyses

All statistical analyses were performed using R software version 2.15.3 [26]. Data were analyzed using analysis of variance (ANOVA) to test the significance of year, tillage and cropping treatments and their interactions for each location separately. When significant, means were separated at *P* ≤ 0.05 using Fisher’s protected least significant difference (LSD). All data were subjected to Fligner-Killeen test (Fligner test) to determine the homogeneity of variance and Q-Q plots developed to assess data normality. Weed counts were log transformed [Log (Y +1)] before statistical analyses.

Agreements about the land use for the purpose of research were signed upon inception of this project between Project Leader, Jay B. Norton from the University of Wyoming, and the leadership of the Manor House Agricultural Centre in Kitale, Kenya, and with the leadership of Farmers Training Centre under the direct supervision from the Kenya Ministry of Agriculture in Mabanga, Kenya. Documents are available for viewing upon request.

## Results

### Weed Density: Manor House and Mabanga

**Fig 2:**
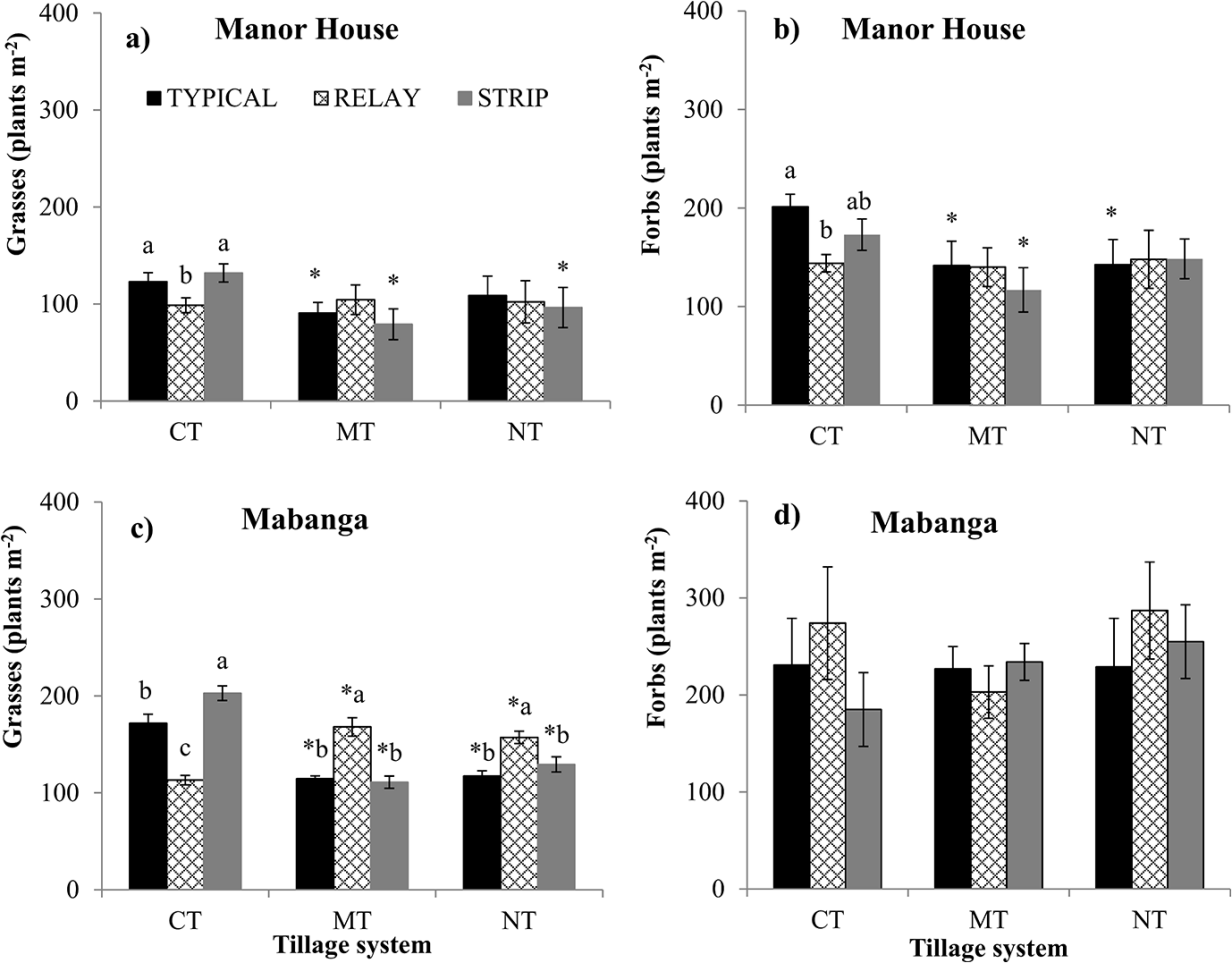
Grass and forb weed density (plants m^-2^) in conventional tillage (CT), minimum tillage (MT) and no-till (NT) systems at Manor House (a and b) and Mabanga (c and d). Error bars represent standard error of the mean (n = 12). Lower case letters represent statistically significant differences between cropping systems within tillage (*P ≤ 0*.*05*). “*” indicates statistically significant difference between tillage systems within cropping (*P ≤ 0*.*05*).

There were fewer weeds at Manor House than at Mabanga (255 versus 364 plants m^-2^) with more forbs than grasses at both locations. At Manor House, grass and forb cover demonstrated significant tillage-by-cropping system and tillage-by-year interactions (Table 1). MT in TYPICAL cropping had 26.0% lower grass cover and MT and NT had 29.0% lower forb cover when compared with CT in TYPICAL cropping (Fig 2A and 2B). CT in RELAY cropping 24.3% lower grass cover and 29.1% lower forb cover when compared with CT in TYPICAL and STRIP cropping.

**Table 1:** F-values for ANOVA of the effect of year, tillage, cropping and their interactions for grass and forb weed counts, Shannon Diversity Index (SDI), maize height and leaf area index (LAI) at Manor-House and Mabanga for 2011–2013. ”*” represents statistical significance at *P ≤ 0*.*05*, “**” represents statistical significance at *P≤ 0*.*01* and “***” represent statistical significance at *P ≤ 0*.*001*.

		Manor House					Mabanga			
Source	DF	Grass	Forb	SDI	Maize height	Maize LAI	Grass	Forb	Maize height	Maize LAI
		——————————————*F value—*———————————-					——————————*F value—————————-*			
Year	2	42.1***	94.6***	0.06	68.4***	110.8***	3.33*	7.30**	20.0***	90.8***
Tillage	2	12.28**	13.66***	11.16**	8.56**	8.81*	1.76	0.16	0.32	0.53
Cropping	2	0.16	1.77	3.69*	0.35	0.66	0.37	1.46	0.61	0.09
Year-by-Tillage	4	13.27***	11.97***	5.76**	4.52**	3.45*	0.23	0.63	0.74	1.08
Year-by- Cropping	4	0.76	2.20	3.53*	2.38*	2.92*	0.84	1.75	1.31	0.98
Tillage-by- Cropping	4	2.31*	2.39*	1.46	1.31	2.56	3.7**	1.01	0.68	1.16
Year-by-Tillage-by-Cropping	8	0.78	0.90	0.42	0.94	0.99	0.62	1.12	0.83	0.65

At Mabanga, only grass cover was significantly affected by a tillage-by-cropping system interaction (Table 1). Grass cover was 44.2% and 34.4% lower under NT and MT in TYPICAL cropping compared with under CT (Fig 2C). Moreover, MT and NT in STRIP cropping and TYPICAL cropping had 30.0% and 22.1% lower grass cover compared with MT and NT in RELAY cropping.

**Fig 3:**
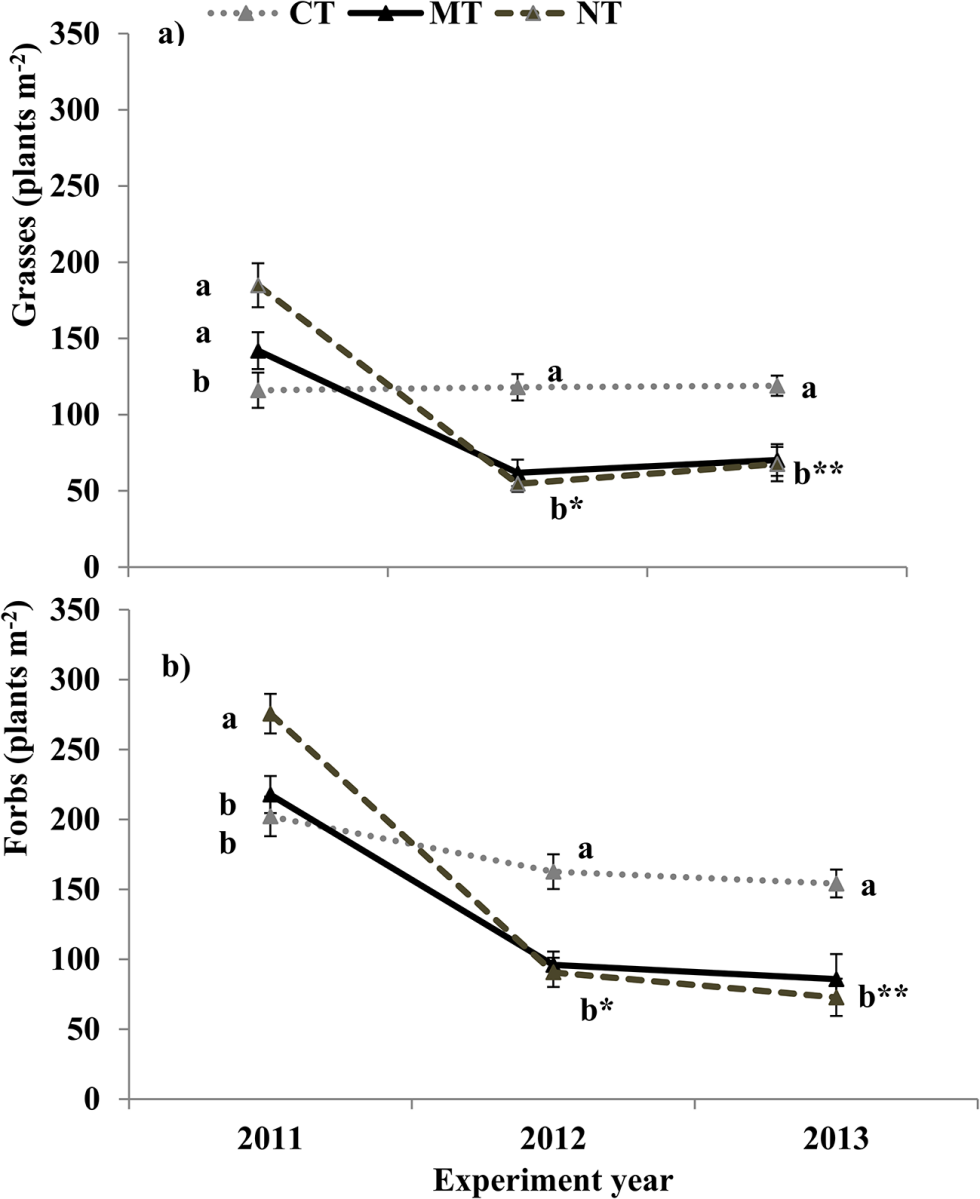
Grass (a) and forb (b) weed density (plants m^-2^) in conventional tillage (CT), minimum tillage (MT) and no-till (NT) systems at Manor House in 2011, 2012 and 2013. Error bars represent standard error of the mean (n = 12). Lower case letters indicate statistical differences between tillage systems within each year (*P ≤ 0*.*05*). “*” indicates statistically significant difference between 2011 and 2012 (*P ≤ 0*.*05*) and “**” indicates statistically significant difference between 2011 and 2013 (*P ≤ 0*.*05*).

At Manor House, there was also a significant year-by-tillage interaction for grass and forb cover (Table 1). In 2011, the highest forb cover was under NT and amounted to 276 weeds m^-2^ followed by MT and CT. Similarly, the highest grass cover amounted to 185 weeds m^-2^ in NT also followed by MT and CT. Weed cover in CT remained unchanged between 2011 and 2013 (Fig 3A and 3B). In 2012, however, NT demonstrated 61.4% decline in grass cover and 72.3% decline in forb cover with no change in forb and grass cover in 2013 (Fig 3A and 3B). Consequently, MT resulted in 51.0% decline in grass cover and 60.0% decline in forb cover between 2011 and 2012 and no change between 2012 and 2013.

### Weed Species Diversity: Manor House

Three out of the nine most abundant weeds were from the *Asteraceae* family. The most frequently observed weeds were *Ageratum conyzoides* (forb) and *Digitaria abyssinica* (grass). Four of the most abundant species were perennial and five were annuals. A number of weed species responded to tillage or cropping system but no significant response to tillage-by-cropping system interaction was observed (Table 2). Tillage did not affect the abundance of *Ageratum conyzoides* in contrast to the significant effect cropping system had. Moreover, *Digitaria abyssinica*, the second most frequently observed weed species, declined under MT but not NT in both STRIP and RELAY cropping. The greatest declines in abundance in response to reducing tillage were observed in *Cyperus rotundus* (grass) and *Commelina benghalensis* and *Richardia brasiliensis* (both forbs). RELAY cropping resulted in declines in *Ageratum conyzoides* (weak response), *Digitaria abyssinica*, *Commelina benghalensis* and *Cyprus rotundus* while STRIP cropping significantly lowered the density of *Ageratum conyzoides* and *Digitaria abyssinica* and *Commelina benghalensis* (weak response) and *Cyperus rotundus* (weak response).

**Table 2:** Scientific name, common name, family, lifecycle, life-form and plant density (plants m^-2^) of most dominant weed species in conventional tillage (CT), minimum tillage (MT) and no-till (NT) and maize/bean intercropping (TYPICAL), maize/bean intercropping relayed with mucuna (RELAY) and maize/bean/mucuna in adjacent strips (STRIP) at Manor House in 2013. Lower case letters represent significant differences between treatments (*P ≤ 0*.*05*). “Ann” stands for “annual” and “Per” stands for “perennial”.

Scientific Name	Common Name	Family	Lifecycle	Lifeform	Tillage System			Cropping System		
					CT	MT	NT	TYPICAL	RELAY	STRIP
					————————————plants m^-2 —^——————————					
*Ageratum conyzoides* L.	Goat Weed	*Asteraceae*	Ann	Forb	69	66	64	84a	66ab	50b
*Digitaria abyssinica* Hochst. Ex A. Rich. & Stapf.	African Couch Grass	*Poaceae*	Per	Grass	45a	30b	44a	54a	34b	31b
*Commelina benghalensis* L	Benghal Dayflower	*Commelinaceae*	Ann	Forb	41a	11b	7b	21a	18b	20ab
*Cyperus rotundus* L.	Nut Grass	*Cyperaceae*	Per	Grass	4a	12b	9b	22a	16b	21ab
*Bidens pilosa* L.	Black-Jack	*Asteraceae*	Per	Forb	34a	22ab	20b	18	24	33
*Oxalis latifolia* Kunth	Woodsorrel	*Oxalidaceae*	Per	Forb	28	17	25	26	21	25
*Richardia brasiliensis* Gomes	Mexican Clover	*Rubiaceae*	Ann	Forb	18a	4b	6b	12	11	6
*Oxygonum sinuatum* Hochst. & Steud. Ex Meisn.	Double Thorn	*Polygonaceae*	Ann	Forb	13	10	5	11	13	5
*Tagetes erecta* L.	African Marigold	*Asteraceae*	Ann	Forb	11	10	5	7	8	9

The Shannon Diversity Index demonstrated significant year-by-tillage and year-by- cropping system interactions (Table 1). In general, CT had the highest SDI of 0.75 in both years. The second highest SDI of 0.66 was under NT in 2012 but the values were comparable between MT and NT in 2013 (Table 3). Significant year-by-cropping interaction was observed in 2012 only when STRIP cropping had the highest SDI of 0.72 and TYPICAL cropping had the lowest SDI of 0.61 (Table 3).

**Table 3:** Shannon Diversity in conventional tillage (CT), minimum tillage (MT) and no-till (NT) and maize/bean intercropping (TYPICAL), maize/bean intercropping relayed with mucuna (RELAY) and maize/bean/mucuna in adjacent strips (STRIP) at Manor House in 2012 and 2013. Lower case letters indicate statistical differences between different tillage and cropping systems within a year (*P ≤ 0*.*05*). “*” indicates significant differences between years within the same tillage or cropping system (*P ≤ 0*.*05)*.

	Shannon Diversity Index	
	2012	2013
**Tillage**		
**CT**	0.77a	0.72a
**MT**	0.57b	0.68ab
**NT**	0.66c	0.58ab*
**Cropping**		
**TYPICAL**	0.72a	0.70
**RELAY**	0.68ab	0.60
**STRIP**	0.61b	0.69*

### Costs of Weed Control: Manor House

Costs associated with land preparation for planting were the highest in CT ($266.00 ha^-1^) and relied solely on animal drawn and hand hoe tillage operations (Table 4). In contrast, costs of land preparation and planting in MT and NT relied on herbicides and hand hoe or panga, which amounted to $50.00 ha^-1^. Costs associated with weed management after planting were $216.00 ha^-1^ in CT and were $67.60 ha^-1^ and $66.40 ha^-1^ higher than in MT and NT, respectively. Costs of herbicides during this time amounted to $103.00 ha^-1^ in MT and $136.00 ha^-1^ in NT while costs of labor associated with herbicide application amounted to $73.00 ha^-1^ in MT and $143.00 ha^-1^ in NT. Total costs of weed management amounted to $333.60 ha^-1^ in MT, $332.40 ha^-1^ in NT and $482.00 ha^-1^ in CT.

**Table 4:** Production costs associated with weed control during land preparation and after planting in conventional tillage (CT), minimum tillage (MT) and no-till (NT) at Manor House obtained during long rainy season.

			COSTS								
			CT			MT			NT		
Management	Mode/*Active Ingredient*	Freq./*Rate*	Materials	Labor	Total	Materials	Labor	Total	Materials	Labor	Total
			^———————————^US Dollars ha^-1 ———————————^								
***Weed Control during Land Preparation*:**											
											
Tillage	Animal Drawn Moldboard Plow	2x		144.00							
Harrowing	Hand Hoe	1x		72.00	**144.00**						
Planting	Hand Hoe	1x		50.00	**72.00**		50.00	**50.00**			
	Jab Planter	1x			**50.00**					50.00	**50.00**
**TOTAL**	** **	** **	**0.00**	**266.00**	**266.00**	**0.00**	**50.00**	**50.00**	**0.00**	**50.00**	**50.00**
											** **
***Weed Control after Planting*:**											** **
											** **
Tillage	Hand Hoe	2x (CT)		216.00	**216.00**		108.00	**108.00**			** **
Herbicides:		1x (MT)									** **
*Dual Gold*	*S-Metachlor 960 g L* ^*-1*^	*576 g ha* ^*-1*^				54.20	36.50	**90.70**	54.20	36.50	**90.70**
*Touchdown*	*Glyphosate 500 g L* ^*-1*^	*750 g ha* ^*-1*^				48.40	36.50	**84.90**	48.40	36.50	**84.90**
*Basagran*	*Bentazone 400 g L* ^*-1*^	*600 g ha* ^*-1*^							33.80	73.00	**106.80**
**TOTAL**	** **	** **	**0.00**	**216.00**	**216.00**	**102.60**	**181.00**	**283.60**	**136.40**	**146.00**	**282.40**
**GRAND TOTAL**			**0.00**	**482.00**	**482.00**	**102.60**	**231.00**	**333.60**	**136.40**	**196.00**	**332.40**

### Maize Growth: Manor House

Despite large inter-annual variability observed at the two locations, interactions between year-by-tillage and year-by- cropping for LAI and maize height were significant at Manor House (Table 1). In 2011, maize plants in CT were 37.8 cm and 33.3 cm taller than in MT and NT, respectively, while in 2013, maize plants in CT were 20.5 cm taller than in NT (Table 5). Similarly, LAI in CT was consistently greater compared with MT and NT with the largest differences of 1.2 and 0.9 between CT and MT and NT, respectively in 2011 and the smallest differences of 0.6 and 0.4 between CT and MT and NT, respectively in 2013.

**Table 5:** Maize plant height and leaf area index (LAI) at V7-V8 growth stages in conventional tillage (CT), minimum tillage (MT) and no-till (NT) and maize/bean intercropping (TYPICAL), maize/bean intercropping relayed with mucuna (RELAY) and maize/bean/mucuna in adjacent strips (STRIP) at Manor House in 2011, 2012 and 2013. Lower case letters indicate statistical differences between different tillage and cropping systems within a year (*P ≤ 0*.*05*).

Management System	Maize Height			Maize LAI		
	2011	2012	2013	2011	2012	2013
	——————cm——————					
**Tillage**						
**CT**	126.1a	77.5	221.7a	3.7a	2.0a	3.8a
**MT**	88.3b	74.0	205.5ab	2.5b	1.7b	3.2b
**NT**	89.7b	75.3	201.2b	2.8b	1.9ab	3.4b
**Cropping**						
**TYPICAL**	100.4	67.6b	210.3	2.9	1.6b	3.5
**RELAY**	103.2	77.9a	201.7	3.0	2.0a	3.4
**STRIP**	100.5	81.6a	216.5	2.9	1.9a	3.5

In 2012, cropping system appeared to have a significant impact on both maize height and LAI (Table 5). Maize in RELAY cropping was 10.3 cm taller and in STRIP cropping was 14.0 cm taller compared with TYPICAL cropping. Similarly, LAI was 0.4 and 0.3 greater in RELAY and STRIP compared with TYPICAL cropping.

Tillage-by-cropping interaction also significantly influenced LNC (Table 5). The differences however, were observed in CT only. Specifically, CT in RELAY cropping had the highest N content of 33.2 mg g^-1^ (data not presented).

## Discussion

Weed competition is often the main limitation to adoption of CA systems on smallholder farms [27]. Results from this project suggest that introducing minimum till and no-till in western Kenya resulted in immediate declines in grass and forbs populations. Not only did the overall grass and forb cover declined, but the most notorious weeds (two perennial grasses and two annual forbs) showed significant reduction. These findings partially contradicted results from studies by Wrucke and Arnold [28] and Chauhan et al. [29] who observed declines in forb cover but increases in grass cover and attributed the increase in grass cover to the release from competition with forbs.

Reduction in weed density were more pronounced at the higher elevation Manor House site, where crops are grown during one long growing season and managed with fewer tillage operations. In contrast, weaker weed population responses to reducing tillage in lower elevation areas where crops were grown twice per year were also observed by Mandumbu et al. [30]. Gopal et al. [31] observed higher weed density following more frequent tillage in rice production.

Herbicide use was an intricate component of the MT and NT systems in this study. Thus, declines in weed cover during the transition were also attributed to using chemicals, also in line with observations by Nyamangara et al. [32]. Consequently, better understanding of herbicide use, availability at local distribution outlets and smaller packaging can aid with adoption of alternative tillage practices by smallholder farmers. Both MT and NT resulted in weed cover declines in STRIP cropping and *Ageratum conyzoides*, the most abundant weed species, demonstrated the greatest decline also under STRIP cropping. Mono-cropped strips likely facilitated more effective weed control and use of herbicides compared with RELAY or TYPICAL cropping.

Greater declines in weed density in response to reduced tillage rather than new cropping systems confirmed that the effectiveness of using cover crops to control weeds may become evident later in the transition, also proposed by Riemens et al. [33]. Transitioning to RELAY combined with reduced tillage did not affect weed cover except for grass cover increase at Mabanga. Decline in the abundance of the four most dominant weed species in maize planted under RELAY cropping however, was statistically significant, but of much smaller magnitude than changes due to reduced tillage. This observation further indicated that is too early to see the response of cover crop-based CA systems on weed control. It is known that mucuna planted before maize can smother weeds as observed by Ngwira et al. [34] and Ikuenobe and Anoliefo [35], but also terminating mucuna before maize planting may require as much as 15 to 80% more herbicide application, as observed by Andersson and D’Souza [27]. More research on timing and herbicide application using dose response approaches could help farmers manage multiple benefits associated with cover crops.

Monitoring maize height, LAI and LNC at V7-V8 stage helped determine a possibility of plants being stressed during the fast growing vegetative phase. Reducing tillage resulted in staggered plant growth and lower LAI compared with CT, which confirmed earlier observations by Aikins et al.[24]. Conversely, not only were the maize plants taller and had greater LAI in RELAY managed with CT, but the same combination also demonstrated higher LNC compared with other systems. Since no correlation between weed density and maize yields existed, the reduced maize performance at V7-V8 stage in MT and NT were likely due to other factors emerging during the early transition stage that were not measured during this experiment.

Using MT and NT lowered operational and input costs before and after planting by about 30%. Herbicides appeared to significantly reduce costs of otherwise more expensive manual labor or rental of the tillage equipment. Thus reduced- and no-till approaches can be viable options in socio-economic settings where farmers have access and capital to purchase herbicides. It is also a viable option in areas where reducing tillage does not negatively impact crop production due to, for example, high accumulation of clay in the sub-surface soil horizons. Costs of manual labor however, should be considered in relative terms, as some of the work is usually performed by family members, thus reducing the overall cash flow outside of the household. A combination of both reduced tillage and herbicide use may bring the most desirable effects. In addition, under certain circumstances, reducing tillage may result in temporary increases in weed density and increase the demand for manual labor and operational costs, as observed by Baudron et al. [36].

In general, previous understanding of the lack of immediate increases in farm income while transitioning to CA has been often associated with reduced success of CA adoption [6]. Since small-holder farmers generally value short-term returns more than long-term benefits, practices that reduce investments in labor and, ultimately, require fewer chemicals to combat reduced populations of weeds will become beneficial. More detailed economic analyses of production input costs and returns of the entire operation should provide additional information. These research results however, demonstrated the reduction in weed density and population diversity as early as two years into the transition without any negative impacts on maize yield and growth. It is therefore, an important starting point that can guide local research and extension during transition [37]. Such analyses are important to determine robust recommendations designed for specific agro-ecological and socio-economic conditions [32].
